# Nutrition intervention for migraine: a randomized crossover trial

**DOI:** 10.1186/1129-2377-15-69

**Published:** 2014-10-23

**Authors:** Anne E Bunner, Ulka Agarwal, Joseph F Gonzales, Francesca Valente, Neal D Barnard

**Affiliations:** 1 Physicians Committee for Responsible Medicine, 5100 Wisconsin Ave. NW, Suite 400, Washington, DC 20016, USA; 2 California State University, East Bay, Student Health and Counseling Services, 25800 Carlos Bee Boulevard, Hayward, CA 94542, USA; 3 George Washington University School of Medicine, 2150 Pennsylvania Ave., NW, Washington, DC 20037, USA

**Keywords:** Migraine, Headache, Nutrition, Diet, Plant-based, Vegan

## Abstract

**Background:**

Limited evidence suggests that dietary interventions may offer a promising approach for migraine. The purpose of this study was to determine the effects of a low-fat plant-based diet intervention on migraine severity and frequency.

**Methods:**

Forty-two adult migraine sufferers were recruited from the general community in Washington, DC, and divided randomly into two groups. This 36-week crossover study included two treatments: dietary instruction and placebo supplement. Each treatment period was 16 weeks, with a 4-week washout between. During the diet period, a low-fat vegan diet was prescribed for 4 weeks, after which an elimination diet was used. Participants were assessed at the beginning, midpoint, and end of each period. Significance was determined using student’s t-tests.

**Results:**

Worst headache pain in last 2 weeks, as measured by visual analog scale, was initially 6.4/10 cm (SD 2.1 cm), and declined 2.1 cm during the diet period and 0.7 cm during the supplement period (p=0.03). Average headache intensity (0–10 scale) was initially 4.2 (SD 1.4) per week, and this declined by 1.0 during the diet period and by 0.5 during the supplement period (p=0.20). Average headache frequency was initially 2.3 (SD 1.8) per week, and this declined by 0.3 during the diet period and by 0.4 during the supplement period (p=0.61). The Patient’s Global Impression of Change showed greater improvement in pain during the diet period (p<0.001).

**Conclusions:**

These results suggest that a nutritional approach may be a useful part of migraine treatment, but that methodologic issues necessitate further research.

**Trial registration:**

Clinicaltrials.gov, NCT01699009 and NCT01547494.

## Background

Migraine disorder is characterized by headaches with moderate to severe pain, often having a unilateral location and pulsating quality, accompanied by nausea, vomiting, photophobia, or phonophobia [1]. Migraines affect over 28 million Americans and occur at all ages, more often in women than men [2]. Medications have an important role in prevention and treatment but are limited in effectiveness while carrying side effects that may include cardiovascular risks and headaches due to medication overuse. The pathology of migraine is incompletely understood, but evidence suggests dietary factors may contribute, perhaps through inflammation and vasodilation.

Limited evidence suggests that dietary interventions may offer a promising approach for migraine [3]. A low-fat diet has been shown to reduce headache frequency, intensity, and duration, with subsequently lowered medication use [4]. A recent review identified 16 population studies with data on dietary precipitating factors [5]. Greater than 5% of participants identified specific food triggers in 8 of the studies. The most commonly reported triggers in these and other analyses include: chocolate, cheese, citrus, alcohol, and coffee [5,6]. In these articles, triggers were reported retrospectively by patients, but other studies have identified triggers via elimination diets [7].

Some have attempted to identify dietary triggers via blood testing for IgG antibodies. In a small randomized cross-over study, participants’ blood was tested for antibodies against 266 foods. Each participant eliminated foods for which they had antibodies, leading to a 29% reduction in migraine days [8]. A subsequent randomized controlled trial eliminated antibody-promoting foods in the diets of 84 migraineurs [9]. After 4 weeks, this diet was associated with a 19% reduction in headache days as compared with the control “sham” diet. While similar antibody tests do not show predictive value for other indications [10,11], these studies demonstrate the potential of food elimination for reducing pain.

Diet may also affect migraines through more indirect mechanisms. Changes in plasma estrogen concentrations throughout menstrual cycles are strongly associated with migraine [12]. Diet changes, particularly a low-fat, high-fiber, vegan diet, appear to reduce estrogen activity and the intensity and duration of premenstrual symptoms [13]. Therefore, such a diet may be expected to reduce frequency of headaches occurring in the premenstrual period.

These data suggest that a low-fat, vegan diet may be beneficial, combining the advantages of a high-fiber diet and a diet free of animal-derived triggers. To our knowledge, vegan diets have not been tested for therapeutic potential in migraine. We hypothesized that a vegan dietary intervention designed to eliminate potential dietary triggers for migraine would reduce headache frequency and pain.

## Methods

### Participants and recruitment

Individuals with recurrent migraine were recruited from the Washington, DC, area through newspaper and radio advertisements and a letter from a local neurologist. All participants had a prior migraine diagnosis, as defined by the criteria of the second edition of the International Classification of Headache Disorders, [1] experienced migraines at least twice per month, were over 18 years of age, and were following an omnivorous or lacto-ovo vegetarian diet. The study was approved by Ethical and Independent Review Services. A screening interview was conducted to determine eligibility. All participants gave informed consent. No inducements for participation were provided. The study was registered at clinicaltrials.gov with registration numbers NCT01699009 and NCT01547494.

Volunteers were randomly assigned by study staff, using a random-number table, to group 1 or 2 for 36 weeks. Allocation concealment was achieved using unmarked assignment envelopes. It was not possible to blind participants and instructors to group assignment.

### Study design and procedures

**Figure 1:**
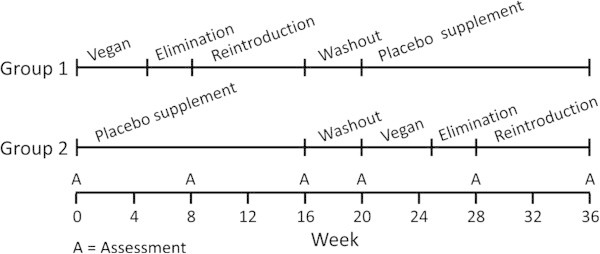
**Study design.** Group 1 did the diet treatment first, followed by the supplement treatment. Group 2 did the two treatments in the reverse order.

We used a crossover design to compare a diet change with a placebo supplement. Participants were randomly assigned either (1) to make dietary changes or (2) to take a placebo supplement and make no diet changes for the first 16 weeks of the study. After a 4-week washout period with no treatment, participants crossed over to the other treatment condition for the last 16 weeks of the study (Figure 1). During the diet period, participants received weekly dietary instruction. A low-fat vegan diet was prescribed for 4 weeks, after which an elimination diet was used to enable participants to identify possible specific pain trigger foods. During the elimination diet period, participants were asked to continue the low-fat vegan diet and also to eliminate common trigger foods, chosen based on previous studies (Table 1) [2,6,14]. Participants were asked to adhere to the elimination diet until no further improvement was noted or until the period midpoint (typically 10–21 days), after which they were asked to reintroduce the omitted foods one at a time, starting with foods least likely to be a trigger, every 48 hours and report subsequent headaches.

**Table 1:** Elimination diet

**Foods to avoid**	**Foods to favor**
Grains:	Grains:
Wheat	Oats
Rye	Rice
Barley	Quinoa
Corn	Buckwheat
	Amaranth
Legumes:	Sorghum
Soybeans	Millet
Chickpeas	Teff
Peanuts	
	Legumes:
Fruits:	Lentils
Citrus fruits (all)	
Bananas	Fruits:
Apples	Pears
	Apricots
Vegetables:	Blueberries
Nightshade family (tomatoes, eggplant, peppers,	Plums
potatoes)	
Allium genus (onions, garlic)	Vegetables:
Sweet potatoes, yams	Artichokes
Celery	Asparagus
	Broccoli, Cauliflower
Other:	Brussels sprouts
Animal products (all)^a^	Cabbage, Bok choy
Nuts and seeds (all)	Carrots
Chocolate	Greens: Chard, Kale, Collards, Mustard, Spinach,
Sugar	Lettuce
Coffee	Zucchini, other hard and soft squashes
Tea	
Alcohol	Condiments:
	Olive oil
	Vanilla extract
	Brown rice syrup
	Maple syrup
	Salt

^a^Participants avoided animal products during entire diet phase, not only during elimination diet.

The placebo supplement was a capsule containing 10 mcg alpha-linolenic acid and 10 mcg vitamin E, packaged in a plain white bottle. This supplement was chosen because it could credibly be presented as potentially having some clinical efficacy, while having no actual effect in the current study. In higher doses, omega-3 oils and vitamin E may have efficacy in inflammatory conditions [15,16]. The participants were told that the supplement contained a mixture of omega-3 oils and vitamin E. Participants were asked to take one capsule daily.

Participants were asked to keep their medications constant to the extent possible, but to follow the advice of their personal physicians.

### Dependent variables

Our primary outcomes were headache frequency as measured by headache diaries and pain severity as measured by a physician-administered visual analog scale (VAS). Assessments occurred at the beginning, midpoint, and end of each 16-week period. To monitor dietary intake, participants completed 2-day diet records and filled out a diet questionnaire that included questions about avoiding foods and consumption frequency. Two-day diet records are a valid means of measuring food and nutrient intakes [17]. Body weight, body mass index (BMI, weight in kilograms divided by height squared), and plasma lipid concentrations were also assessed. Participants rated headache pain using a visual analog scale. Participants were asked to mark along a 10 cm line according to the severity of their worst headache pain in the preceding 2 weeks. The scale was anchored on the left side with the words “No pain” and on the right side with “Pain as bad as it could possibly be”.

At the end of each treatment period, patients were asked to rate symptom change during that period using an 5-point Likert-style scale, ranging from “much worse” to “much better”. In the 2nd replication, this scale was replaced by the patient’s global impression of change (PGIC) question, which measures subjective pain improvement by asking participants to rate symptom change on a scale of 1–7 from “no change” to “a great deal better” [18]. This change was made to utilize a validated scale. Participants also kept a weekly diary of headache number, intensity (numerical rating scale from 1 to 10), and duration during the entire study. Headache frequency was defined as average number of headaches per week. Multiple dimensions of wellness were assessed with the Rand Short Form 36 (SF-36) [19].

### Statistical analysis

Since little has been published on the effects of a vegan diet on pain in migraine patients, a power analysis could not be based on previous research. Therefore, we chose an exploratory approach that did not limit sample size and accepted all volunteers who met the inclusion criteria.

For pain scales, body weight, and lipids (total cholesterol, LDL-cholesterol, HDL-cholesterol, and triglycerides), descriptive statistics were calculated. For normally distributed data, parametric tests for significant effects were used; for non-normally distributed variables, non-parametric tests were used. An alpha of 0.05 was used for all statistical tests. Headache diary data (headache frequency, intensity, duration, and days) were averaged over 4–8 weeks, based on the time between assessments. For example, headache frequency was defined as the average number of headaches per week since the last assessment.

Two-tailed T-tests for independent samples were calculated for the changes during the diet and supplement periods (significance cutoff, 0.05). For the second 16-week period, proximal baseline data (ie, data immediately preceding that period) were used, rather than actual study baseline. For missing data, the most recent available values were brought forward, except for body weight, for which baseline values were used. For each endpoint, 5-9% of datapoints were missing. Participants who dropped out in the first period were deemed to have had no change in any variable during the second period. Both intention-to-treat analysis and completers analyses were performed. For the completers analysis, only participants who attended the baseline and endpoint assessments for both treatments were included. For the multivariate analysis, baseline values for key outcome variables were included as covariates, regardless of significant differences between the two groups at baseline. Data analyses were performed by statisticians who were impartial to the hypotheses being tested.

In addition, due to the complications associated with the crossover design, a subanalysis of all variables was performed on the first 16 weeks of data.

## Results

### Recruitment

**Figure 2:**
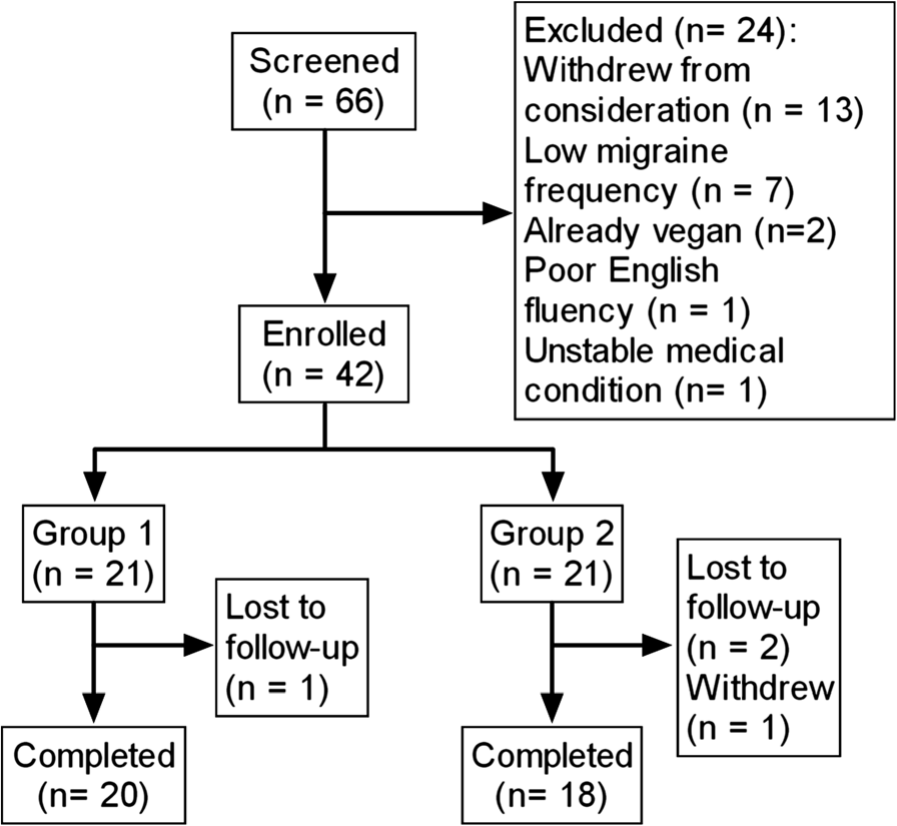
**Recruitment and flow of participants through trial.** Study completers attended final assessment at 36 weeks. All enrolled participants were included in the analysis.

Sixty-six participants were interviewed and 42 (mean age 46 ± 13) were enrolled (Figure 2). Recruitment periods were December 2011-January 2012 and October 2012-January 2013. Participants were 93% women, well-educated (95% college degree or higher), 83% white, and 90% non-Hispanic (Table 2). Among the 26 participants reporting an onset date for their migraines, the mean disease duration was 24 years. At baseline, 4 participants met the criteria for chronic migraine (15 or more headache days per month), while the rest met the criteria for episodic. There were no significant demographic or clinical differences between the groups. Thirty-eight participants (90%) completed the study (Figure 2). Most participants reported following an omnivorous diet at baseline, with 12% (N=5) following an ovo-lacto vegetarian diet. None avoided all animal products at baseline.

**Table 2:** Baseline characteristics

**Variable**	**All participants**^**a**^	**Group 1 (N=21)**	**Group 2 (N=21)**	**p value**^**b**^
Women N	39	19 (90%)	20 (95%)	0.55
College degree or higher N	40	20 (95%)	20 (95%)	0.32
White race N	35	18 (86%)	17 (81%)	0.68
Hispanic N	4	3 (14%)	1 (5%)	0.29
Nonvegetarian N	37	18 (86%)	19 (90%)	0.63
	Mean (SD)	Mean (SD)	Mean (SD)	p value^c^
Age (y)	45.7 (12.7)	49 (13.5)	42.4 (11.1)	0.09
Weight (kg)	76.9 (19.8)	78.0 (22.6)	75.7 (17.1)	0.71
BMI	27.6 (6.0)	27.7 (6.4)	27.5 (5.8)	0.90
Illness duration (y) (N=26)	23.7 (12.6)	24.7 (13.8)	22.7 (11.7)	0.69
Total cholesterol (mg/dl)	191.1 (36.9)	195.6 (42.5)	186.9 (31.1)	0.46
VAS (cm) (N=41)	6.4 (2.1)	6.1 (2.4)	6.7 (1.9)	0.38
Headache number (per wk) (N=40)	2.3 (1.8)	2.1 (1.6)	2.6 (2.1)	0.50
Headache intensity, scale 0–10 (N=37)	4.2 (1.4)	3.9 (1.1)	4.5 (1.7)	0.20
Headache duration, hrs (N=38)	5.8 (3.4)	6.5 (3.3)	5.1 (3.5)	0.19
Headache days (per wk) (N=40)	2.2 (1.4)	2.1 (1.6)	2.3 (1.2)	0.64

*Abbreviations: BMI* Body Mass Index, *VAS* visual analog pain scale, worst pain last 2 weeks, *SD* standard deviation.

^a^N=42 unless otherwise indicated.

^b^P values from Chi squared tests for differences between groups.

^c^P values from Student’s T-tests for differences between groups.

There were no significant study-related adverse effects. The study ended as per the protocol.

### Diet adherence

On diet records conducted for 2 days at the diet intervention period midpoint and again for 2 days at the end of the diet period, 28 of the 42 participants reported no consumption of animal products, while 7 reported consumption of at least modest amounts of dairy products or egg ingredients and 3 reported consumption of at least modest amounts of meat during one of these diet-record assessments. Diet adherence was not assessed for the 4 drop-outs. Food frequency questionnaire data confirmed the findings of the diet records. Because the elimination diet was individualized and participants were free to implement it as they wished, it was not possible to assess adherence to the elimination diet.

### Changes on clinical measurements

In our intention-to-treat analysis, we combined data from all participants for each period. Body weight fell by 3.6 kg during the diet period, and 0.1 kg during the supplement period (p< 0.001, Table 3). Total cholesterol fell by 14 mg/dL during the diet period, but increased by 1 mg/dL during the supplement period (p=0.03). LDL cholesterol fell by 9 md/dL during the diet period, but increased by 2 mg/dL during the supplement period (p=0.04). For some variables, change scores showed non-normality in at least one comparison group, but non-parametric tests yielded results that did not meaningfully differ from those of the parametric tests. The completer analysis yielded essentially the same results as the intention-to-treat analysis (Table 4).

**Table 3:** Diet effects on clinical measures, intention-to-treat analysis

	**Diet period**			**Supplement period**			**Effect Size**^**e**^	**p value**^**f**^
	**Baseline**	**16 weeks**	**Change**	**Baseline**	**16 weeks**	**Change**		
	**Mean (SD)**	**Mean (SD)**	**Mean (SD)**	**Mean (SD)**	**Mean (SD)**	**Mean (SD)**	**Mean (SD)**	
Body weight (kg) (N=42)	76.7 (20.2)	73.1 (19.4)	-3.6 (3.8)^a^	74.9 (19.7)	74.8 (19.9)	-0.1 (2.4)	-3.5 (4.4)	< 0.001
BMI (N=42)	27.5 (6.2)	26.2 (5.9)	-1.3 (1.3)^a^	26.9 (6.0)	26.8 (6.1)	0.0 (0.9)	-1.3 (1.6)	< 0.001
Total cholesterol (N=41)^g^	189.5 (38.8)	175.8 (42.7)	-13.7 (32.0)^c^	184.7 (33.6)	185.4 (38.0)	0.7 (26.2)	-14.4 (40.5)	0.03
HDL (N=42)	61.1 (15.7)	55.3 (14.3)	-5.8 (15.2)^d^	61.9 (14.1)	60.7 (14.3)	-1.2 (10.0)	-4.6 (19.0)	0.12
LDL (N=41)	108.8 (33.6)	99.8 (34.5)	-9.0 (26.3)^d^	103.1 (28.3)	105.5 (32.7)	2.3 (22.7)	-11.1 (33.6)	0.04
Ratio (N=41)	3.2 (0.9)	3.3 (0.9)	0.1 (0.8)	3.1 (0.7)	3.2 (0.8)	0.1 (0.7)	-0.1 (1.2)	0.75
Triglycerides (N=41)	95.9 (44.3)	102.7 (42.1)	6.8 (51.9)	96.3 (35.1)	96.2 (51.7)	-0.1 (49.2)	6.9 (77.6)	0.57
Log triglycerides (N=41)	1.94 (0.20)	1.98 (0.18)	0.04 (0.21)	1.96 (0.16)	1.94 (0.18)	-0.01 (0.15)	0.05 (0.28)	0.25
VAS (cm) (N=41)	6.0 (2.7)	3.8 (3.0)	-2.1 (3.2)^b^	4.9 (2.8)	4.2 (2.8)	-0.7 (2.3)	-1.4 (4.0)	0.03
Headache number (per wk) (N=40)^h^	2.1 (1.4)	1.8 (1.8)	-0.3 (1.2)	2.2 (2.1)	1.8 (2.2)	-0.4 (0.9)	0.1 (1.3)	0.61
Headache intensity (N=37)	4.3 (1.9)	3.3 (1.9)	-1.0 (1.7)^c^	3.8 (2.0)	3.3 (2.0)	-0.5 (1.5)	-0.5 (2.5)	0.20
Headache duration, hrs (N=38)	6.2 (4.0)	5.2 (4.0)	-0.9 (2.9)	5.2 (3.9)	4.8 (4.0)	-0.3 (2.5)	-0.6 (4.4)	0.44
Headache days (per wk) (N=40)	2.0 (1.4)	1.8 (1.5)	-0.3 (1.1)	2.1 (1.7)	1.7 (1.6)	-0.4 (0.8)	0.1 (1.3)	0.62
Medicated headache number (N=36)	1.4 (1.1)	1.1 (1.5)	-0.2 (1.5)	1.4 (1.5)	1.1 (1.1)	-0.3 (0.8)	0.0 (1.7)	0.90
% headaches medicated (N=36)	65.1 (33.5)	46.0 (32.7)	-19.2 (39.3)^c^	52.6 (32.4)	49.4 (34.4)	-3.2 (21.2)	-16.0 (48.5)	0.04

*Abbreviations: BMI* Body Mass Index, *HDL* high-density lipoprotein cholesterol, *LDL* low-density lipoprotein cholesterol, Ratio= Total cholesterol/HDL, *VAS* visual analog pain scale, worst pain last 2 weeks, *SD* standard deviation.

^a^p<0.0001, ^b^p<0.001, ^c^p<0.01, ^d^p<0.05, all from within-group T-tests.

^e^Effect size is difference between diet treatment effect and supplement treatment effect.

^f^p value is from between group T-test.

^g^One participant with incomplete blood lipids data was excluded from analyses of total, HDL, and LDL cholesterol and triglycerides. Cholesterol and triglycerides are reported in mg/dl.

^h^Variable Ns reflect the fact that participants with incomplete headache diary data were excluded from analysis of headache number, intensity, duration, days, and/or medication rates as appropriate.

**Table 4:** Diet effects on clinical measures, study completers only

	**Diet period**			**Supplement period**			**Effect Size**	**p value**^**e**^
	**Baseline**	**16 weeks**	**Change**	**Baseline**	**16 weeks**	**Change**		
	**Mean (SD)**	**Mean (SD)**	**Mean (SD)**	**Mean (SD)**	**Mean (SD)**	**Mean (SD)**	**Mean (SD)**	
Body weight (kg) (N=36)^f^	75.0 (18.7)	71.1 (17.3)	-3.9 (3.8)^a^	73 (17.7)	72.9 (18.1)	0.0 (2.6)	-3.9 (4.6)	<0.001
BMI (N=36)	26.9 (5.7)	25.5 (5.2)	-1.4 (1.4)^a^	26.2 (5.4)	26.2 (5.6)	0.0 (1.0)	-1.4 (1.6)	<0.001
Total cholesterol (N=35)^g^	185.9 (36.8)	169.3 (35.5)	-16.5 (32.8)^c^	181.2 (31.7)	181.3 (36.4)	0.1 (25.4)	-16.7 (41.5)	0.02
HDL (N=36)	60.9 (16.5)	54.6 (15.0)	-6.3 (16.1)^d^	60.9 (14.7)	60.4 (14.8)	-0.6 (10.4)	-5.7 (19.9)	0.09
LDL (N=35)	105.5 (32.1)	94.2 (27.9)	-11.3 (27)^d^	100.5 (26.2)	101.7 (30.9)	1.2 (20.4)	-12.2 (33.6)	0.04
Ratio (N=35)	3.2 (1.0)	3.2 (0.9)	0.0 (0.9)	3.1 (0.7)	3.1 (0.8)	0.1 (0.7)	0.0 (1.2)	0.85
Triglycerides (N=35)	95.2 (46.4)	101.9 (43.5)	6.7 (56)	96.6 (36.6)	96.5 (55.0)	-0.1 (53.2)	6.7 (83.7)	0.64
Log triglycerides (N=35)	1.93 (0.2)	1.97 (0.18)	0.04 (0.23)	1.95 (0.17)	1.94 (0.19)	-0.01 (0.16)	0.05 (0.30)	0.30
VAS (cm) (N=35)	6.0 (2.7)	3.6 (3.0)	-2.4 (3.2)^a^	4.7 (2.8)	4.1 (2.8)	-0.6 (2.3)	-1.8 (3.9)	0.01
Headache number (per wk) (N=35)	2.1 (1.4)	1.7 (1.9)	-0.3 (1.2)	2.1 (2.0)	1.8 (2.2)	-0.4 (0.9)	0.0 (1.3)	0.95
Headache intensity (N=33)	4.3 (1.8)	3.1 (1.8)	-1.2 (1.7)^b^	3.6 (1.9)	3.2 (2.0)	-0.4 (1.5)	-0.8 (2.4)	0.07
Headache duration (N=33)	6.1 (4.1)	4.8 (3.6)	-1.3 (2.7)^c^	4.5 (3.2)	4.4 (3.6)	-0.1 (2.4)	-1.2 (3.9)	0.08
Headache days (per wk) (N=35)	2.0 (1.4)	1.7 (1.6)	-0.3 (1.1)	1.9 (1.6)	1.6 (1.6)	-0.3 (0.7)	0.0 (1.2)	0.99
Number medicated (N= 35)	1.4 (1.1)	1.1 (1.6)	-0.3 (1.6)	1.2 (1.2)	1.1 (1.1)	-0.2 (0.7)	-0.1 (1.7)	0.73
% medicated (N= 35)	65.2 (31.8)	41.3 (31.3)	-23.8 (38.8)^b^	50.1 (32.3)	46.1 (33.6)	-4.0 (22.5)	-19.8 (49.7)	0.02

*Abbreviations: BMI* Body Mass Index, *HDL* high-density lipoprotein cholesterol, *LDL* low-density lipoprotein cholesterol, Ratio= Total cholesterol/HDL, *VAS* visual analog pain scale, worst pain last 2 weeks, *SD* standard deviation.

^a^p<0.0001; ^b^p<0.001; ^c^p<0.01; ^d^p<0.05.

^e^p values are from between-group T-tests.

^f^Participants who attended the baseline and endpoint assessments for both treatments included here.

^g^Cholesterol and triglycerides are reported in mg/dl.

### Changes in reported pain

**Figure 3:**
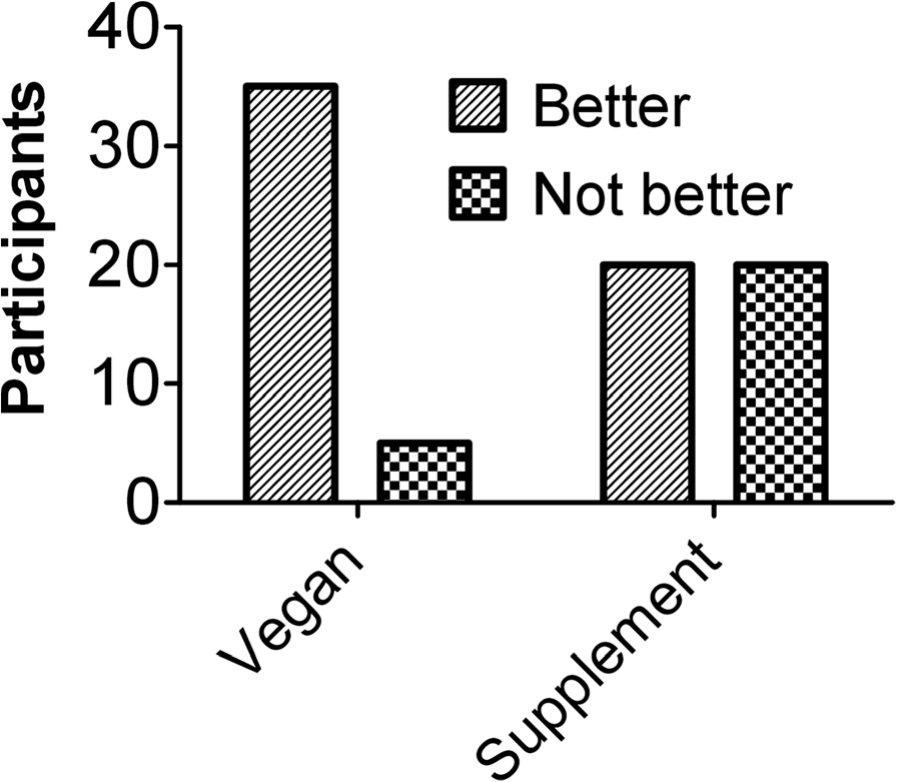
**Change in pain.** Number of participants in each treatment reporting improvement (better; a little better, but no noticeable change; somewhat better, but the change has not made a real and worthwhile difference; moderately better, and a slight but noticeable change; better, and a definite improvement that has made a real and worthwhile difference) or no improvement (no change [or condition has got worse]; almost the same, hardly any change at all; worse; much worse) on the PGIC or change in pain questions.

Severity of worst pain in the preceding 2 weeks, as measured by a visual analog scale, showed significantly more improvement during the diet period than the supplement period (-2.1 cm [35%] vs -0.7 cm [14%], p=0.030, Table 3). Pain improvement as measured by the PGIC or change in pain question was significantly greater after the diet period, with 35 of 40 participants describing their symptoms as “better” after the diet period, and 5 reporting no improvement, while participants were evenly split after the supplement period, with 20 reporting improvement and 20 reporting no improvement (p<0.001, Figure 3, Additional file 1: Table S1).

Non-parametric tests on non-normal data confirmed the above results, except that decreases in number of headaches (p=0.04) and number of medicated headaches (p=0.004) were significant during the diet period (Table 3).

In a completers analysis, significant decreases in headache intensity, duration, and percent headaches requiring pain relief medication were observed during the diet period (all p<0.01), and in headache number and days during the supplement period (both p<0.03, Table 4). A significant diet effect was observed for VAS (p=0.01) and percent headaches medicated (p=0.02).

Differences in migraine prevention medication changes were not significant. A subanalysis with only completers who did not change their migraine prevention medications (N= 26) showed significant decreases in headache intensity and the percent of headaches requiring pain relief medication during the diet period as compared to the control (both p<0.03).

Frequency of pain relief medication use fell by 19 absolute percentage points (p=0.004) during the diet period and 3 absolute percentage points during the supplement period (Table 3). Headache intensity from diaries declined by 1.0 point on a 0–10 scale during the diet period, but only 0.5 points in the supplement period, although this difference was not significant. The differences in the change scores for headache number, intensity, duration, and days did not reach significance.

### Changes in mood

In an intention-to-treat analysis, the SF-36 data showed significant increases in social functioning (p=0.01) and in physical functioning, role limitations due to physical health, energy/fatigue, emotional well-being, pain, and general health (all p<0.03), during the diet period, although between-period differences did not reach statistical significance (Additional file 1: Table S2). Pain rated on the SF-36 improved by 17 points in the diet period, but also improved by 13 points in the supplement period.

### Order effects

For group 1, the supplement period followed the diet period. Many of these participants declined to return to their baseline diets at the end of the period, despite the study requirement that they do so. Thirty percent (N=6) of these participants reported eating meat or fish less than once a month or not at all during the washout and the entire supplement period. An additional 2 participants reported avoiding meat and fish for at least the 4-week washout period.

Because of this incomplete cross-over, although clinical characteristics of the two groups were not significantly different at baseline (Table 1), some were significantly different at the start of the respective supplement periods (week 0 for group 2, week 20 for group 1). Body weight, VAS, and headache intensity changed in group 1 during the diet period and did not return to baseline levels during the washout period (Additional file 1: Table S3).

A subanalysis of the headache data examining only the first 16 weeks of the study was therefore conducted to examine data free of the potentially confounding effects of the incomplete crossover. This analysis showed significant decreases in headache number, intensity, days, and number and percent medicated headaches in the diet group (all p<0.05, Table 5). Modest improvements in headache frequency, days, and number and percent medicated headaches were also observed in the supplement group. Although between-group differences favored the diet group in each case, these differences were significant only for changes in headache intensity.

**Table 5:** Diet effects on clinical measures, analysis of first 16 weeks

	**Diet group (N=21)**			**Supplement group (N=21)**			**Effect size**	**p value**^**c**^
	**Baseline**	**16 weeks**	**Change**	**Baseline**	**16 weeks**	**Change**		
	**Mean (SD)**	**Mean (SD)**	**Mean (SD)**	**Mean (SD)**	**Mean (SD)**	**Mean (SD)**	**Mean (95% CI)**	
Body weight (kg)^d^	78.0 (22.6)	73.8 (22.1)	-4.3 (4.5)^a^	75.7 (17.1)	75.3 (18.1)	-0.4 (2.9)	-3.9 (-6.2 to -1.5)	0.002
BMI	27.7 (6.4)	26.2 (6.3)	-1.5 (1.7)^a^	27.5 (5.8)	27.3 (6.1)	-0.2 (1.1)	-1.4 (-2.2 to -0.5)	0.003
Total cholesterol^e^	195.6 (42.5)	173.6 (48.9)	-22.0 (41.7)^b^	186.9 (31.1)	185.6 (37.2)	-1.3 (30.0)	-20.7 (-43.6 to 2.1)	0.07
HDL	62.2 (17.6)	57.3 (17.1)	-4.9 (19.1)	62.9 (12.9)	58.8 (12.1)	-4.1 (9.5)	-0.8 (-10.2 to 8.6)	0.86
LDL	111.0 (35.4)	95.5 (38.8)	-15.5 (34.4)	104.9 (27)	107.2 (32.7)	2.4 (27.0)	-17.8 (-37.3 to 1.7)	0.07
Ratio	3.3 (1.0)	3.1 (0.8)	-0.1 (1.0)	3.1 (0.7)	3.3 (0.9)	0.2 (0.8)	-0.3 (-0.9 to 0.3)	0.25
Triglycerides	107.8 (41.2)	101.1 (35.5)	-6.7 (53.0)	95.5 (38.2)	98.1 (68.3)	2.6 (61.5)	-9.3 (-45.6 to 27.1)	0.61
Log triglycerides	2.01 (0.15)	1.98 (0.16)	-0.03 (0.21)	1.95 (0.18)	1.93 (0.23)	-0.02 (0.16)	0.0 (-0.1 to 0.1)	0.85
VAS (cm)	6.1 (2.4)	3.0 (2.9)	-3.1 (3.0)^a^	6.7 (1.9)	6.0 (2.0)	-0.7 (2.4)	-2.4 (-4.1 to -0.7)	0.007
Headache number (per wk)	2.1 (1.6)	1.6 (1.7)	-0.5 (1.0)^b^	2.6 (2.1)	2.3 (2.4)	-0.3 (1.0)	-0.2 (-0.9 to 0.4)	0.46
Headache intensity	3.9 (1.1)	2.7 (1.8)	-1.2 (1.8)^b^	4.5 (1.7)	4.5 (2)	0.0 (1.5)	-1.2 (-2.3 to -0.1)	0.04
Headache duration, hrs	6.5 (3.3)	5.2 (4.4)	-1.3 (3.0)	5.1 (3.5)	5.1 (2.8)	0.0 (2.3)	-1.6 (-3.5 to 0.4)	0.15
Headache days (per wk)	2.1 (1.6)	1.6 (1.6)	-0.4 (0.9)^b^	2.3 (1.2)	2.1 (1.5)	-0.2 (0.9)	-0.3 (-0.8 to 0.3)	0.38
Medicated headache number	1.6 (1.3)	1 (1.3)	-0.6 (0.7)^a^	1.4 (0.8)	1.3 (0.9)	-0.2 (0.7)	-0.5 (-0.9 to 0.01)	0.06
% headaches medicated	61.8 (28.5)	44.3 (30.5)	-17.5 (37.8)^b^	66.1 (29.6)	64.1 (32.5)	-1.9 (24.1)	-15.5 (-36.1 to 5)	0.13

*Abbreviations: BMI* Body Mass Index, *HDL* high-density lipoprotein cholesterol, *LDL* low-density lipoprotein cholesterol, Ratio= Total cholesterol/HDL, *VAS* visual analog pain scale, worst pain last 2 weeks, *SD* standard deviation.

^a^p<0.001; ^b^p<0.05, for within-group T-tests.

^c^p value is from between group T-test.

^d^For specific N values, see Table 3.

^e^Cholesterol and triglycerides are reported in mg/dl.

A multivariate analysis showed that VAS and headache intensity at 16 weeks were significantly, positively associated with number of headaches at baseline. The effect of the diet remained significant after adjustment.

## Discussion

In a crossover design, a vegan diet was associated with greater reductions in reported pain, as measured by VAS, PGIC when compared with changes during the supplement period. In addition, during the diet period, significant changes were observed in headache intensity, pain as measured by SF-36, as well as in body weight, and total, LDL, and HDL cholesterol.

Previous studies have shown that a low-fat diet can improve migraine pain [4]. In addition, elimination diets have demonstrated effectiveness in several studies [3,8,9]. We observed a smaller change in headache days as compared to a previous study excluding IgG antibody-eliciting foods [8]. This may be due to the duration of the elimination diet intervention (6 weeks as opposed to our 2–3 weeks), the extent of the dietary restriction imposed on patients, or the methods of food reintroduction.

### Potential mechanisms

There are a number of possible mechanisms by which a vegan and trigger-free diet could reduce pain. Evidence suggests an involvement of neurogenic inflammation and neurogenic vasodilation in migraine [20]. Many plant foods are high in antioxidants and anti-inflammatory compounds. In addition, a vegan diet excludes certain commonly reported migraine triggers (ie, dairy products). Meat products have been shown to have inflammatory properties [21,22], and eliminating these might be expected to have an anti-inflammatory effect. Some meats and cheeses are high in tyramine, which has been linked to migraine [2].

The benefits of weight loss for migraine have been demonstrated elsewhere [23,24]. Indeed, a recent paper on weight loss and migraine showed symptom improvement in a group following a low-calorie diet and a group following a ketogenic very-low-calorie diet [25]. Therefore, it is possible that the pain reducing effects of the vegan diet may be, at least in part, due to weight reduction. In addition, the possibility that lower blood pressure or hormonal changes [13], which commonly occur with plant-based diets [13,26] may, in turn, favorably influence migraine symptoms, cannot be ruled out.

In this study, some improvements in pain and clinical measurements were observed during the supplement period. Pain, as measured by the SF-36, improved, as did headache number and headache days. These changes may have been in part due to incomplete crossover for group 1 (diet then supplement group), placebo, or seasonal effects for both groups.

The study has several strengths. An advantage of dietary interventions that include community volunteers who are not confined or restricted is that the results readily translate into real-life applications. This protocol maximized dietary adherence by using group support and frequent monitoring of reported dietary intake.

The study also has limitations. The self-reported nature of pain is an inherent limitation of headache studies. Migraine triggers unrelated to diet, such as stress and weather changes, cannot be controlled and may have influenced the weekly headache diary data. Designing a placebo control for diet studies is challenging. The placebo supplement may not have been an ideal control for the diet, since the process of a diet change is different than the process of adding a daily supplement. However, it would not have been credible to ask control participants to eliminate an arbitrary list of foods, so the nutritional compound was deemed to be the most appropriate control available.

The effects of the diet cannot be separated from the effects of the weekly classes. The SF-36 showed the diet was associated with improved social functioning, and this may have been an effect of the classes, of reduced pain, or both.

Although the intervention was intended to combine the vegan diet with the elimination of additional foods that may have been potential triggers, the individualized nature of the elimination period meant that it was challenging to separate the effects of the vegan diet *per se* from those of the elimination period. In addition, some individuals had not fully reintroduced all eliminated foods by study’s end. Therefore, it is not possible to attribute the reported changes in pain specifically to either the elimination or vegan phase. A longer study, permitting a fuller elimination and reintroduction period, could isolate those variables.

Many individuals were unwilling to return their previous diets at the conclusion of the diet phase. Similarly, prior crossover studies have reported that individuals trying plant-based diets often refuse to abandon them, despite protocol requirements [13,27]. While this observation suggests some substantial benefit of the dietary intervention, it created a methodologic problem in that the diets and some outcome measures for group 1 participants were different from group 2 at the start of the supplement period and that participants crossing into the supplement period may continue to benefit from the previous dietary intervention that they never fully abandoned. The net result of these effects would be an apparent reduction in the observed effectiveness of the dietary intervention.

Appropriately planned vegan diets are nutritionally adequate for all life stages, and pose no additional risk for patients [28]. In addition, a vegan diet presents advantages in reduced risk for diabetes and heart disease, among other conditions [29,30].

## Conclusions

These results suggest potential value of a nutritional approach to migraine treatment. Further studies are needed to enable dietary pain triggers to be isolated and to confirm the usefulness of the vegan diet as compared to alternative therapeutic diets. A longer trial, separating the effects of a vegan intervention from a diet eliminating additional foods would be helpful, if the considerable technical barriers to such a study can be overcome.

## Abbreviations

BMI: Body mass index; HDL-cholesterol: High-density lipoprotein cholesterol; LDL-cholesterol: Low-density lipoprotein cholesterol; IgG: Immunoglobulin G; PGIC: Patient’s global impression of change; SD: Standard deviation; SF-36: Rand Short Form 36; VAS: Visual analog pain scale.

## Competing interests

The authors declare that they have no competing interest.

## Authors’ contributions

NDB designed the study. UA, JFG, and FV acquired the data. AEB analyzed the data and wrote the manuscript. NDB revised the manuscript. All authors read and approved the final manuscript.

## Authors’ information

NDB is the founder and president of the Physicians Committee for Responsible Medicine, a nutrition research and advocacy non-profit.

## Supplementary Material

Additional file 1: Table S1 Change in pain. **Table S2.** 36-Item short form survey for general health, intention-to-treat analysis. **Table S3.** Outcomes analyzed for order effects on supplement period. Click here for file
