# Supplementary material for: Nutrition intervention for migraine: a randomized crossover trial
Source: J Headache Pain. 2014 Oct 23;15(1):69. doi: 10.1186/1129-2377-15-69 (PMC4227630; doi:10.1186/1129-2377-15-69)
Supplement: Additional file 1: Table S1 — Change in pain. Table S2. 36-Item short form survey for general health, intention-to-treat analysis. Table S3. Outcomes analyzed for order effects on supplement period. [file 1129-2377-15-69-S1.doc]

**Nutrition Intervention for Migraine: a Randomized Crossover Trial**

Additional file 1

Table S1. Change in pain

Table S2. 36-Item short form survey for general health, intention-to-treat analysis

Table S3. Outcomes analyzed for order effects on supplement period

**Table S1**. Change in pain

| **Description** | **Diet period** | **Supplement period** |
| --- | --- | --- |
| No change (or condition has got worse); almost the same, hardly any change at all; worse; much worse | 5 | 20 |
| Better; A little better, but no noticeable change; Somewhat better, but the change has not made a real and worthwhile difference; Moderately better, and a slight but noticeable change; Better, and a deﬁnite improvement that has made a real and worthwhile difference | 27 | 13 |
| Much better; A great deal better, and a considerable improvement that has made all the difference | 8 | 7 |
| p value from chi-squared test | | <0.001 |

Collapsed data from PGIC and change in pain question.

**Table S2**. 36-Item short form survey for general health, intention-to-treat analysis

|  | **Diet period** | | | **Supplement period** | | | **p value^e^** |
| --- | --- | --- | --- | --- | --- | --- | --- |
|  | **Baseline** | **16 weeks** | **Change** | **Baseline** | **16 weeks** | **Change** |  |
|  | **Mean (SD)** | **Mean (SD)** | **Mean (SD)** | **Mean (SD)** | **Mean (SD)** | **Mean (SD)** |  |
| Physical functioning^f^ | 88 (13) | 93 (9) | 93 (9)^c^ | 89 (17) | 91 (11) | 2 (9) | 0.13 |
| Role limitations due to physical health | 60 (39) | 78 (36) | 78 (36)^c^ | 77 (35) | 82 (30) | 5 (35) | 0.08 |
| Role limitations due to emotional problems | 77 (36) | 86 (30) | 86 (30) | 82 (35) | 83 (32) | 1 (36) | 0.36 |
| Energy/fatigue | 52 (21) | 61 (20) | 61 (20)^c^ | 54 (18) | 55 (20) | 1 (17) | 0.06 |
| Emotional well being | 75 (13) | 79 (12) | 79 (12)^c^ | 73 (14) | 74 (13) | 1 (12) | 0.24 |
| Social functioning | 78 (23) | 86 (17) | 86 (17)^d^ | 82 (24) | 81 (20) | -1 (20) | **0.03** |
| Pain | 62 (28) | 72 (22) | 72 (22)^c^ | 65 (20) | 74 (20) | 9 (18)^c^ | 0.69 |
| General Health | 62 (19) | 66 (19) | 66 (19)^d^ | 62 (19) | 64 (18) | 3 (12) | 0.71 |

a, p < 0.0001; b, p < 0.001; c, p < 0.01; d, p < 0.05.

e, p values are from between-group T-tests

f, N = 42.

**Table S3. Outcomes analyzed for order effects on supplement period**

|  | **Baseline prior to supplement period, group 1 (wk 20)** | **Baseline prior to supplement period, group 2 (wk 0)** | **p value^b^** |
| --- | --- | --- | --- |
|  | **Mean (SD)** | **Mean (SD)** |  |
| Body weight (kg) (N = 42) | 74.1 (22.3) | 75.7 (17.1) | 0.79 |
| BMI (N = 42) | 26.3 (6.3) | 27.5 (5.8) | 0.53 |
| Total cholesterol (N = 41) | 182.5 (36.8) | 186.9 (31.1) | 0.68 |
| HDL (N = 42) | 61 (15.5) | 62.9 (12.9) | 0.68 |
| LDL (N = 41) | 101.4 (30.1) | 104.9 (27) | 0.70 |
| Ratio (N = 41) | 3.1 (0.7) | 3.1 (0.7) | 0.96 |
| Triglycerides (N = 41) | 97.2 (32.4) | 95.5 (38.2) | 0.88 |
| Log triglycerides (N= 41) | 1.97 (0.14) | 1.95 (0.18) | 0.69 |
| VAS (cm) (N = 41) | 3.1 (2.4) | 6.7 (1.9) | **< 0.001** |
| Headache number (per wk) (N = 40)40) | 1.9 (2) | 2.6 (2.1) | 0.30 |
| Headache intensity (N = 37) | 3.1 (2) | 4.5 (1.7) | **0.03** |
| Headache duration (N = 38) | 5.2 (4.4) | 5.1 (3.5) | 0.89 |
| Headache days (per wk) (N = 40) | 1.9 (2) | 2.3 (1.2) | 0.45 |
| Number medicated headaches (per wk) (N = 40) | 1.2 (1.8) | 1.4 (0.8) | 0.64 |
| Percent medicated headaches (N = 40) | 42 (29.9) | 66.1 (29.6) | **0.01** |

Abbreviations: BMI, Body Mass Index; HDL, high-density lipoprotein cholesterol; LDL, low-density lipoprotein cholesterol; Ratio = Total cholesterol/ HDL; VAS, visual analog pain scale, worst pain last 2 weeks; SD, standard deviation.

a, cholesterol and triglycerides in mg/dl.

b, p values are from between-group T-tests
